# From Diet to Dysbiosis: How Nutritional Factors Shape Gut Microbiota and Drive Airway Inflammation in Pediatric Asthma

**DOI:** 10.3390/nu18152496

**Published:** 2026-08-02

**Authors:** Oana Raluca Temneanu, Adriana Mihai, Raluca Olariu, Mihaela Oros, Ileana Ioniuc, Vasile Valeriu Lupu, Ancuța Lupu, Alice Grudnicki, Manuel Florin Roșu, Roxana Șerban, Andreea-Luciana Avasiloaiei, Paula Popovici

**Affiliations:** 1 Grigore T. Popa University of Medicine and Pharmacy, 700115 Iași, Romania; temneanu.oana@umfiasi.ro (O.R.T.); raluca.olariu@umfiasi.ro (R.O.); ileana.ioniuc@umfiasi.ro (I.I.); vasile.lupu@umfiasi.ro (V.V.L.); ancuta.ignat1@umfiasi.ro (A.L.); alice.azoicai@umfiasi.ro (A.G.); florin.rosu@umfiasi.ro (M.F.R.); roxana-n-serban@umfiasi.ro (R.Ș.); andreea.avasiloaiei@umfiasi.ro (A.-L.A.); paula.popovici@umfiasi.ro (P.P.); 2 Titu Maiorescu University, 031593 Bucharest, Romania; mihaela.oros@prof.utm.ro

**Keywords:** gut microbiota, gut–lung axis, paediatric asthma, dietary patterns, dysbiosis, short-chain fatty acids, airway inflammation, nutrition, butyrate, obesity-related asthma

## Abstract

Asthma prevalence in school-age children varies widely by geography, from below 5% in some regions to above 20% in others, averaging 10–12% across industrialized countries, but genetic factors account for less than 40% of disease liability. Gut microbiota is increasingly recognized as a critical intermediary between early-life dietary exposure and immunological trajectories that determine asthma susceptibility. Through the production of short-chain fatty acids (SCFAs), particularly butyrate, propionate, and acetate, commensal bacteria regulate dendritic cell function, promote T-regulatory (Treg) cell differentiation, and attenuate Th2-polarized airway inflammation via the gut–lung axis. Epidemiological cohorts including CHILD, WHEALS, and PASTURE associate early-life dysbiosis and reduced *Lactobacillus*, *Bifidobacterium*, and *Faecalibacterium prausnitzii* with increased asthma risk, while dietary patterns rich in fermentable fibre, omega-3 polyunsaturated fatty acids, and diverse plant-based foods are associated with preserved microbial diversity and, in preclinical models, attenuated type-2 inflammatory signalling. This narrative review synthesizes mechanistic and epidemiological evidence on how nutritional exposures shape gut microbiota composition and influences asthma onset and severity in paediatric populations, including the distinct obesity-related asthma phenotype. Critical gaps, insufficient dietary intervention trials with microbiome endpoints, methodological heterogeneity across cohorts, and the paucity of data from non-Western populations are discussed, with implications for preventive nutritional counselling in paediatric allergology practice.

## 1. Introduction

Methods: This narrative review draws on the literature indexed in PubMed, Scopus, and Web of Science. Search terms combined “gut microbiota”, “gut microbiome”, “gut–lung axis”, “short-chain fatty acids”, “SCFA”, “butyrate”, “dietary fiber”, “dietary pattern”, “Mediterranean diet”, “omega-3”, “vitamin D”, “probiotics”, “prebiotics”, “paediatric asthma”, “childhood asthma”, “obesity-related asthma”, “airway inflammation”, “dysbiosis”, “Th2 inflammation”, “FeNO”, “CHILD cohort”, “PASTURE”, and “WHEALS”. Priority was given to publications from January 2000 to May 2026, with foundational mechanistic studies published earlier included where relevant. Reference lists of pertinent reviews were screened for additional sources. As a narrative review, the selection of studies was guided by relevance to the diet, microbiota, and airway inflammation axis rather than by a predefined systematic protocol, and no formal risk-of-bias assessment was applied.

Asthma affects an estimated 262 million individuals worldwide, and childhood-onset disease accounts for the largest fraction of this burden [1]. Prevalence is strikingly heterogeneous geographically; while point prevalence in school-age children across high-income countries averages 10–12%, the underlying range spans from below 5% in parts of Eastern Europe and Asia to above 20% in some Anglophone and Latin American urban centres [1,2]. This variability is itself informative, it tracks with dietary Westernization, reduced microbial exposure, and environmental pollution rather than with genetic background, and it foreshadows the central argument of this review. Low- and middle-income countries are reporting rising incidence without the diagnostic infrastructure needed to manage it.

Genetics explains, at best, 35–40% of disease liability, a figure that has not shifted substantially over decades of genomic inquiry despite the identification of more than 100 susceptibility loci [3]. The residual variance implicates modifiable environmental exposures operating from conception onward, among which diet, by determining gut microbiota composition, may represent the most tractable single target. The intestinal microbiome actively educates the neonatal immune system, calibrates Th1/Th2 balance during the critical early-life window, and sustains barrier integrity at both the intestinal and pulmonary mucosal surfaces. When dietary inputs are insufficient in diversity, fermentable fibre, and prebiotic substrate, the microbial community undergoes a shift that includes reduced alpha-diversity, contraction of butyrate-producing taxa, and relative expansion of pro-inflammatory species, collectively termed dysbiosis [4]. The downstream immunological consequences include impaired Treg induction, amplified Th2 responses, and heightened IgE-mediated sensitization, constituting the core signature of atopic asthma.

This mechanistic cascade has been formalized as the gut–lung axis, a bidirectional communication network linking intestinal microbial metabolism to pulmonary immune homeostasis through metabolite trafficking, enteric nervous system signalling, and systemic cytokine gradients [5]. The growing body of evidence relating to this axis was recently synthesized by Boulund (2025), integrating population data, in vivo models, and interventional trials to map the chain from early-life microbiome disruption to asthma onset [6]. The present review builds on this framework to examine specifically the nutritional determinants of gut microbiota composition and their downstream effects on paediatric airway inflammation, with emphasis on translating mechanistic evidence into clinical and research priorities.

This narrative review synthesizes current evidence regarding the diet → microbiota → airway inflammation axis in children. We examine the mechanistic foundations of the gut–lung axis, characterize the dietary patterns and specific nutrients most consistently linked to microbiota modulation, critically evaluate the epidemiological and interventional data connecting gut dysbiosis to paediatric asthma, including the distinct obesity-related phenotype, and identify the research priorities needed to move the field from association to preventive intervention.

## 2. The Gut–Lung Axis: Mechanistic Foundations

### 2.1. Short-Chain Fatty Acids as Immunological Effectors

**Figure 1:**
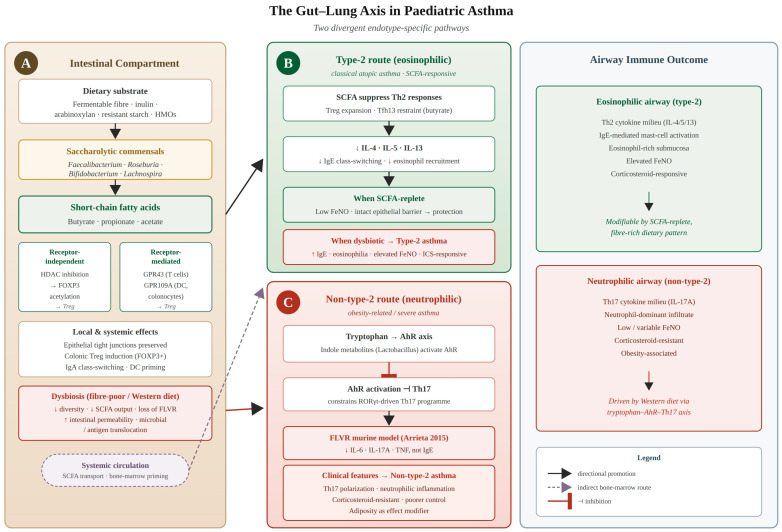
The gut–lung axis in paediatric asthma, showing two divergent endotype-specific pathways across three panels: (**A**) the intestinal compartment, in which dietary substrate is fermented by saccharolytic commensals to short-chain fatty acids; (**B**) the type-2 (eosinophilic) route, which is SCFA-responsive; and (**C**) the non-type-2 (neutrophilic) route, which engages the tryptophan–AhR–Th17 axis. Solid arrows denote directional promotion, the dashed arrow indicates the indirect bone-marrow route, and ⊣ denotes inhibition. Original schematic prepared by the authors. Abbreviations: AhR, aryl hydrocarbon receptor; DC, dendritic cell; FeNO, fractional exhaled nitric oxide; FLVR, Faecalibacterium–Lachnospira–Veillonella–Rothia; FOXP3, forkhead box P3; GPR, G-protein-coupled receptor; HDAC, histone deacetylase; HMO, human milk oligosaccharide; ICS, inhaled corticosteroid; IgA/IgE, immunoglobulin A/E; IL, interleukin; SCFA, short-chain fatty acid; Tfh13, T follicular helper type 13 cell; Th, T helper cell; TNF, tumour necrosis factor; Treg, regulatory T cell.

The mechanistic core of the gut–lung axis rests on the capacity of intestinal bacteria to produce bioactive metabolites that reach systemic circulation and exert immunomodulatory effects at distant mucosal sites (Figure 1). SCFAs including acetate, propionate, and butyrate are the most extensively characterized mediators. Generated through anaerobic fermentation of dietary fibre by saccharolytic bacteria of the Clostridiales order, including *Roseburia intestinalis*, *Eubacterium rectale*, and *Faecalibacterium prausnitzii*, SCFAs signal through G-protein-coupled receptors (GPR41/FFAR3, GPR43/FFAR2, GPR109a/HCAR2) expressed on colonic epithelial cells, immune cells, pulmonary dendritic cells, and the airway epithelium [7,8].

Figure 1 summarizes the architecture developed across this section. Fermentation of dietary fibre and human milk oligosaccharides by saccharolytic commensals yields SCFAs, which act locally to preserve barrier integrity and induce regulatory T cells (Treg) and systemically, through haematopoietic reprogramming of dendritic-cell precursors, to shape airway immune tone. Two divergent pathways follow, distinguished by endotype. In the type-2 (eosinophilic) route, characteristic of classical atopic asthma, SCFAs suppress Th2 responses and IgE, including via butyrate-mediated inhibition of Tfh13 cells; this route responds to inhaled corticosteroids and is the principal target of SCFA-based dietary intervention. In the non-type-2 (neutrophilic) route, characteristic of obesity-related and severe asthma, the tryptophan–aryl hydrocarbon receptor (AhR) axis modulates Th17 responses; consistent with this, the FLVR (Faecalibacterium, Lachnospira, Veillonella, Rothia) murine model of Arrieta et al. reduced Th1/Th17 cytokines rather than eosinophilia (Section 2.4). Each component of this scheme is substantiated in the text that follows.

Butyrate exerts its pro-tolerogenic effects through at least two mechanistically distinct routes that should not be conflated. Regarding receptor-independent effects (HDAC inhibition), as a histone deacetylase (HDAC) inhibitor, butyrate enhances acetylation at the *Foxp3* locus, promoting Treg differentiation from naive CD4^+^ precursors. Receptor-mediated signalling (GPR43/GPR109A) is a parallel and kinetically distinct route that operates through GPR43 (FFAR2) on T cells and GPR109A (HCAR2) on dendritic cells and colonocytes, transducing extracellular butyrate into anti-inflammatory signalling independently of the epigenetic mechanism above. Arpaia et al. (2013) demonstrated that colonic Treg induction by butyrate was CNS1-dependent and operated substantially through HDAC inhibition [9], while Furusawa et al. (2013) confirmed the direct relationship between luminal butyrate and colonic Treg numbers [10]. Distinguishing the epigenetic (HDAC) from the receptor-mediated (GPR43/GPR109a) mechanism matters because they have different dose–response characteristics and different therapeutic implications.

Propionate acts through a mechanism that is frequently misrepresented in the literature. Trompette et al. (2014) demonstrated that the protective effect of dietary fibre operates not primarily through direct action on mature pulmonary dendritic cells, but through GPR41-dependent modulation of haematopoiesis in the bone marrow; a high-fibre diet altered the developmental programming of dendritic cell and macrophage precursors, generating effector cells with an attenuated capacity to promote Th2 responses upon migration to the lung [7]. This distinction, between reprogramming of haematopoietic precursors and direct action on terminally differentiated effector cells, is mechanistically substantive and is conflated in much of the secondary literature.

A more recently characterized pathway involves butyrate-mediated suppression of the Tfh13 cell population. Tfh13 cells, first defined by Gowthaman et al. (2019), constitute a transcriptionally distinct T follicular helper subset with an unusual cytokine profile (IL-13^hi^IL-4^hi^IL-5^hi^IL-21^lo^) co-expressing BCL6 and GATA3; they are required for the production of high-affinity, anaphylaxis-inducing IgE and are detectable in the circulating compartment of food- and aeroallergen-sensitized patients but not in healthy controls [11]. Building on this, Yu et al. (2025) demonstrated in a murine asthma model and a human validation cohort that microbiota-derived butyrate suppressed Tfh13 differentiation and reduced high-affinity IgE [12]. While these findings require independent replication in larger human cohorts, they offer a mechanistically distinct pathway by which dietary fibre deficiency may sustain IgE-mediated atopic disease.

It would be incomplete to present SCFAs as uniformly anti-inflammatory; t#heir effects are dose- and context-dependent. Acetate, in particular, has been shown in murine models to exert both protective and, under certain timing and concentration conditions, pro-inflammatory or Th2-amplifying effects, and the developmental window of exposure modulates the direction of the response. The prevailing model of SCFA-mediated protection is well supported for early-life butyrate and propionate, but the literature does not support a simplistic interpretation in which all SCFAs are protective under all conditions. This nuance is relevant when considering supplementation strategies, where dose and timing may prove decisive.

### 2.2. Barrier Integrity: Intestinal and Pulmonary

The gut–lung axis operates not only through soluble metabolites but through structural mechanisms governing mucosal barrier integrity. A healthy gut microbiota, dominated by butyrate-producing commensals, maintains tight junction protein expression (claudin-1, occludin, ZO-1) in the intestinal epithelium, preventing translocation of microbial fragments and unprocessed dietary antigens into systemic circulation [13]. Dysbiosis disrupts this barrier in a quantifiable manner. Intestinal permeability, measured by lactulose/mannitol ratios, is elevated in children with atopic asthma compared with non-atopic controls, and correlates with serum LPS-binding protein concentrations, a surrogate marker of microbial translocation [14].

The pulmonary epithelium shares structural and functional analogies with the intestinal barrier: tight junctions, mucin secretion, and pattern recognition receptor expression including TLR2, TLR4, and NOD2 [15]. Airway epithelial cells respond to circulating SCFAs via GPR41/43, and SCFA depletion, as occurs in fibre-poor dietary states, correlates with reduced airway barrier function and amplified IL-33 and TSLP release following inhaled allergen challenge in murine models and human bronchial epithelial cell cultures [16]. Within the broader respiratory–microbiome interaction reviewed by Huffnagle et al. (2017), an inverse relationship between gut microbial alpha diversity (Shannon index, which integrates richness and evenness) and FeNO has been described, though the evidence base for a direct quantitative relationship in children remains limited [17].

### 2.3. Secondary Bile Acids, Tryptophan Metabolites, and Emerging Pathways

Beyond SCFAs, the gut microbiota modulates systemic immune tone through additional metabolite classes. Secondary bile acids, deoxycholic acid and lithocholic acid, produced by microbial 7α-dehydroxylation of primary bile acids, activate TGR5 receptors on innate lymphoid cells, macrophages, and natural killer T cells, with downstream suppression of IL-4 and IL-13 production in murine pulmonary tissue [18]. Arrieta et al. (2015) identified secondary bile acid alterations in the urinary metabolome of high-risk infants, with reduced glycolithocholate and tauroursodeoxycholate excretion at three months, suggesting that early microbial bile acid processing contributes to the risk-associated metabolic signature [4].

**Figure 2:**
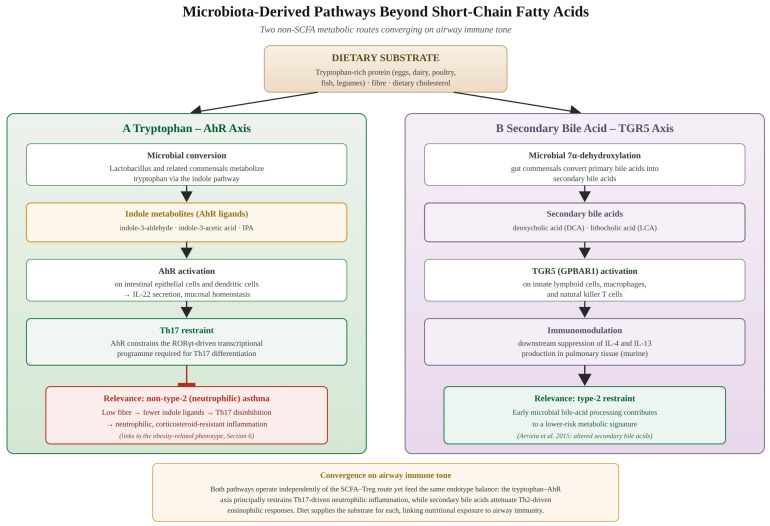
Microbiota-derived metabolic pathways beyond short-chain fatty acids [4]. Two non-SCFA routes are shown. (**A**) The tryptophan–aryl hydrocarbon receptor (AhR) axis: Lactobacillus and related commensals convert dietary tryptophan into indole metabolites that act as AhR ligands on epithelial and dendritic cells, promoting IL-22 secretion and constraining the RORγt-driven Th17 programme, a route of particular relevance to non-type-2 (neutrophilic) asthma. (**B**) The secondary bile acid–TGR5 axis: microbial 7α-dehydroxylation generates deoxycholic and lithocholic acid, which activate TGR5 (GPBAR1) on innate lymphoid cells, macrophages, and natural killer T cells, with downstream suppression of IL-4 and IL-13 in pulmonary tissue. Both pathways operate independently of the SCFA–Treg route and supply the substrate-dependent link between diet and airway immunity. Arrows denote directional promotion and ⊣ denotes inhibition. Original schematic prepared by the authors. Abbreviations: AhR, aryl hydrocarbon receptor; DCA, deoxycholic acid; GPBAR1, G-protein-coupled bile acid receptor 1; IL, interleukin; LCA, lithocholic acid; RORγt, RAR-related orphan receptor gamma t; SCFA, short-chain fatty acid; TGR5, Takeda G-protein receptor 5; Th17, T helper type 17 cell; Treg, regulatory T cell.

Tryptophan metabolites produced by *Lactobacillus* species via the indole pathway activate the aryl hydrocarbon receptor (AhR) on intestinal epithelial cells and dendritic cells, promoting IL-22 secretion and mucosal homeostasis [19]. Critically, AhR signalling also suppresses Th17 differentiation, a pathway of particular relevance to non-type-2 (neutrophilic) asthma endotypes, as discussed in Section 4.2. These non-SCFA pathways remain less well characterized in paediatric asthma, and their integration into a unified mechanistic model is a priority for future multi-omic studies. The supply of substrate for this pathway is diet-dependent; tryptophan-rich protein sources (eggs, dairy, poultry, fish, and legumes) provide the precursor, while the fermentable-fibre matrix sustains the indole-producing Lactobacillus and related commensals that generate these AhR ligands (Figure 2).

### 2.4. The Critical Window: Microbial Colonization in the First 1000 Days

The developmental trajectory of the gut microbiome is acutely time-sensitive. Microbial colonization begins perinatally, earlier in vaginally delivered than caesarean-born infants, and the neonatal community is shaped sequentially by delivery mode, antibiotic exposure, breastfeeding duration, and the timing and diversity of complementary food introduction [20]. The first 1000 days, spanning conception to approximately the second birthday, constitute the critical window during which microbial diversity expands and stabilizes, and during which the immune system acquires its long-term tolerogenic or pro-inflammatory set-point.

Clinical and epidemiological evidence. The CHILD cohort study provided the foundational human evidence. Arrieta et al. (2015) followed 319 infants and stratified them at one year into atopy (*n* = 87), wheeze (*n* = 136), wheeze with atopy (*n* = 22), and control (*n* = 74) groups, with the Asthma Predictive Index assessed at three years [4]. The 22 children with both wheeze and atopic sensitization, the highest-risk group, were 21.5 times more likely to have physician-diagnosed asthma by age three (95% CI 2.4–196.0; *p* = 0.002), the wide confidence interval reflecting the small subgroup size [4]. Arrieta et al. identified four genera, *Faecalibacterium*, *Lachnospira*, *Veillonella*, and *Rothia* (the FLVR consortium), whose combined abundance in the first 100 days of life was selectively reduced in high-risk infants and which normalized thereafter, defining a narrow temporal window. This identification was at the genus level, the resolution limit of the 16S rRNA sequencing employed; species- and strain-level attributions require shotgun metagenomics or targeted qPCR.

Preclinical evidence. A nuance of this landmark study is frequently overlooked and merits emphasis. When FLVR taxa were transplanted into germ-free mice colonized with the dysbiotic microbiota of a high-risk infant, the resulting attenuation of airway inflammation in the offspring was not mediated by reduced eosinophilia or IgE. Rather, FLVR supplementation reduced neutrophil and lymphocyte infiltration and lowered the Th1/Th17-associated cytokines IL-6, IL-17A, and TNF, a profile the authors explicitly noted resembled severe, neutrophilic human asthma rather than the classical Th2-eosinophilic phenotype [4]. The mechanistic implication, discussed further in Section 4.2, is that early microbial deficiency may predispose specifically toward non-type-2 inflammation, a reframing with direct relevance to the obesity-related phenotype. The limitations inherent to germ-free models nonetheless preclude direct causal extrapolation to human disease.

## 3. Dietary Patterns and Gut Microbiota in Children

### 3.1. Mediterranean vs. Western Dietary Patterns

Population-based studies consistently identify dietary pattern, rather than any single nutrient, as the primary ecological determinant of gut microbiota diversity. The Mediterranean dietary pattern, characterized by high intakes of vegetables, legumes, whole grains, fruit, extra-virgin olive oil, and oily fish, with low intake of red meat and ultra-processed foods, is associated with greater alpha diversity, higher relative abundance of butyrate-producing genera, and higher faecal butyrate and propionate concentrations compared with Western patterns [21,22]. Where species-level claims are made (for example, regarding *Prevotella copri* or *Faecalibacterium prausnitzii*), these derive from the subset of studies employing metagenomic rather than 16S amplicon methods.

With specific reference to children, Garcia-Marcos et al. (2013) conducted a systematic review and meta-analysis of eight studies and reported that Mediterranean diet adherence was associated with reduced current wheeze (OR 0.85; 95% CI 0.75–0.98) and severe wheeze (OR 0.66; 95% CI 0.48–0.90), driven principally by Mediterranean-region populations [23]. These pooled estimates require cautious interpretation; both this and subsequent meta-analyses report substantial between-study heterogeneity (I^2^ > 50% for several outcomes), reflecting differences in diet scoring, wheeze definitions, and age ranges. Kouvari et al. (2022) confirmed a protective role across 12 studies while noting that effects on lung function and bronchial hyperresponsiveness were less consistent [24]. Poursoleiman et al. (2024), in a study of 7667 Iranian schoolchildren, found 32% lower odds of recent wheezing in the highest adherence quartile (OR 0.68; 95% CI 0.51–0.90) [25]. The Western pattern produces the inverse microbiome profile: reduced diversity, expansion of *Bacteroides* at the expense of *Prevotella*, and lower SCFA production capacity per unit substrate [26].

Beyond aggregate diversity, the Mediterranean pattern appears to act on the specific microbial functions most relevant to airway immunity. The pattern’s defining components map onto distinct microbial outputs: extra-virgin olive oil polyphenols (oleuropein, hydroxytyrosol) and the fibre of legumes and whole grains together sustain *Faecalibacterium prausnitzii* and *Roseburia*, the principal butyrate producers, while the high ratio of monounsaturated to saturated fat limits the bile-acid shifts that favour pro-inflammatory taxa. The omega-3 content of oily fish, addressed mechanistically in Section 5.1, reinforces this profile. A practical corollary follows for paediatric counselling; the protective signal attaches to the pattern as an ecological whole rather than to any isolated nutrient, which is consistent with the difficulty of reproducing dietary-pattern effects through single-nutrient supplementation, as the negative fibre trial of Rank et al. (2026) illustrates [27]. This distinction matters when translating epidemiological associations into advice, since it argues for whole-diet modification over targeted supplements for the prevention of atopic disease.

### 3.2. Breastfeeding, HMOs, and Early Microbiome Programming

Human milk is increasingly understood as a biologically active system rather than a static source of nutrients, an ecological framing advanced by the BEGIN (Breastmilk Ecology: Genesis of Infant Nutrition) initiative [28]. Human milk oligosaccharides (HMOs), 5–15 g/L in mature milk, are selectively fermented by *Bifidobacterium longum* subsp. *infantis* (a subspecies-level distinction validated by metagenomic and HMO-utilization-gene studies, not by 16S alone), driving the characteristic *Bifidobacterium*-dominated microbiome of breastfed infants [29]. This profile is associated with higher faecal acetate, reduced pathogen abundance, and lower fecal calprotectin compared to formula-fed counterparts. HMOs also directly modulate immune cell function independent of prebiotic activity; 2′-fucosyllactose and lacto-N-tetraose reduce IL-8 secretion by intestinal epithelial cells and promote TGF-β production by dendritic cells ex vivo [29].

Exclusive breastfeeding for at least four months consistently reduces early wheezing risk; its effect on asthma at school age is more attenuated and partly confounded by reverse causation. Roduit et al. (2014), in the PASTURE/EFRAIM study, demonstrated that greater food diversity in the first year was inversely associated with food sensitization at 4.5 and six years [30]. The proposed mechanism involves HMO-mediated microbiome programming followed by diversification-induced expansion of microbial substrate diversity, consistent with the “old friends” hypothesis reformulated mechanistically [31].

The mechanistic basis of HMO bioactivity has been clarified considerably by recent work. More than 200 structurally distinct oligosaccharides have been catalogued, and their function is structure-dependent rather than uniform. Fricker et al. (2025), in work from the Bode laboratory, quantitatively linked individual maternal HMO concentrations to the taxonomic and functional capacity of the infant gut microbiome, and showed that maternal adiposity alters the HMO profile in ways that influence infant microbial function, a finding that connects the breastfeeding and obesity strands of this review [32]. The selective advantage that HMOs confer on *Bifidobacterium longum* subsp. *infantis* is now understood at the level of dedicated transport and glycosidase gene clusters; Wong et al. (2024) detailed how the M-63 strain metabolizes fucosylated and sialylated HMOs and translates this capacity into measurable infant-health benefits [33], while the broader structure-function landscape has been synthesized as the basis of what some authors term the gold standard of infant nutrition [34]. Notably, the microbiome response is not confined to high intakes. Bajic et al. (2024) demonstrated measurable shifts in microbial composition at low predicted daily HMO doses, with implications for the formulation of supplemented formulas [35], and the maternal-milk-to-infant-outcome chain has recently been mapped as a defined HMO–microbiome axis [36]. The anti-infective dimension of this system, spanning pathogen-adhesion inhibition, antibiofilm activity, microbiome modulation, and epithelial reinforcement, has been reviewed in detail elsewhere by our group [37]; for the present purpose it is sufficient to note that the same HMO-driven *Bifidobacterium*-dominated ecology that resists enteric pathogens also establishes the SCFA-producing community implicated in airway immune tolerance.

### 3.3. Delivery Mode, Formula Feeding, and Microbial Colonization

**Figure 3:**
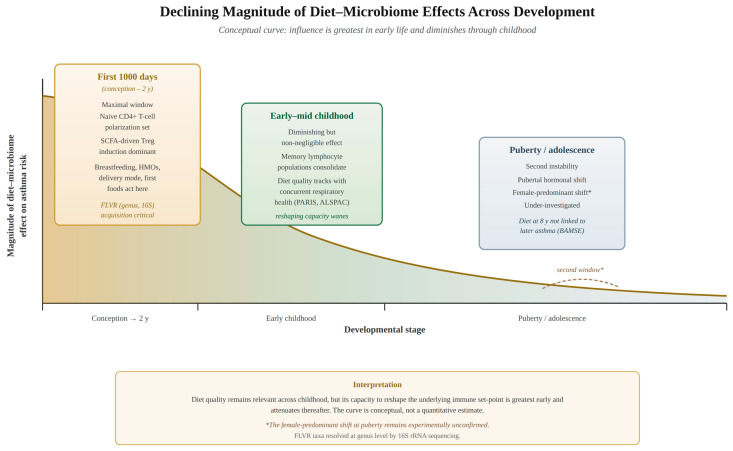
The declining magnitude of diet–microbiome effects on asthma risk across development, greatest during the first 1000 days and diminishing through childhood. * The female-predominant shift at puberty remains experimentally unconfirmed. The curve is conceptual, not a quantitative estimate. Original schematic prepared by the authors. Abbreviations: ALSPAC, Avon Longitudinal Study of Parents and Children; CD4, cluster of differentiation 4; FLVR, Faecalibacterium–Lachnospira–Veillonella–Rothia; HMO, human milk oligosaccharide; SCFA, short-chain fatty acid; Treg, regulatory T cell; 16S, 16S ribosomal RNA gene.

Delivery mode is among the earliest determinants of microbial colonization and the one most consistently linked to later airway disease. Vaginally delivered neonates acquire a maternal faecal and vaginal inoculum dominated by Bifidobacterium and Bacteroides, whereas caesarean-born infants show delayed Bifidobacterium acquisition, transient enrichment of skin- and environment-derived taxa, and relative expansion of Enterobacteriaceae. A meta-analysis of 35 cohort studies found a modest but consistent increase in childhood asthma after caesarean delivery (OR 1.18), with the association concentrated in female offspring (OR 1.26) and absent in males (OR 1.07) [38], a sex difference that parallels the female-predominant pattern noted in Figure 3. The effect size is small relative to the dietary-pattern associations discussed above, and with regard to the maternal conditions that prompt caesarean delivery, confounding by indication cannot be fully excluded.

Critically, the risk appears to follow not from the perinatal perturbation itself but from its persistence. In the COPSAC2010 cohort, Stokholm et al. (2020) followed 700 children and found that caesarean delivery raised asthma risk only in those whose gut microbiota at one year of age still retained the caesarean signature; where the community had matured toward the vaginally delivered profile by twelve months, no excess risk was detectable [39]. This reframes delivery mode within the critical-window model described in Section 2.4; the perinatal inoculum sets an initial trajectory, but the first-year diet that follows determines whether the community converges on a tolerogenic, SCFA-producing configuration.

Feeding mode acts on the same axis and frequently compounds delivery mode, since caesarean birth is associated with delayed breastfeeding initiation, lower rates of exclusive breastfeeding, and earlier transition to formula. Formula-fed infants lack the HMO substrate that drives Bifidobacterium longum subsp. infantis dominance and consequently exhibit lower faecal acetate, higher stool pH, and a more rapid shift toward an adult-like community than breastfed peers [29]. Two strategies measurably attenuate this deficit. In a randomized double-blind trial, supplementation of formula with the synthetic prebiotic mixture scGOS/lcFOS (9:1) together with Bifidobacterium breve M-16V restored timely bifidobacterial colonization, lowered stool pH, and reduced Enterobacteriaceae in caesarean-born infants toward the vaginally delivered reference [40]. As discussed further in Section 5.3, formula supplemented with the HMOs 2′-fucosyllactose and lacto-N-neotetraose shifts the formula-fed microbiome closer to the breastfed pattern. Neither reproduces breastfeeding, but both indicate that the colonization deficit associated with caesarean delivery and formula feeding is at least partially modifiable through early dietary substrate.

### 3.4. Dietary Fibre: Substrate Deficiency and the Limits of Supplementation

Dietary fibre constitutes the primary substrate for colonic SCFA production. Fermentable fibres, inulin-type fructans, fructooligosaccharides, arabinoxylan, and resistant starch stimulate the growth of *Bifidobacterium*, *Lactobacillus*, *Roseburia*, and *Faecalibacterium prausnitzii*, increasing butyrate and propionate output [41]. Paediatric fibre intake is chronically insufficient; median intakes in European children aged 4–10 years range from 10 to 14 g/day against EFSA recommendations of 14–20 g/day.

Trompette et al. (2014) showed in a murine model that a high-fibre diet tripled circulating propionate, expanded pulmonary Treg populations, and attenuated allergen-induced eosinophilia and IgE production, with effects abrogated in germ-free animals [7]. Theiler et al. (2019) demonstrated that SCFA-deficient environments upregulated airway epithelial permeability markers and amplified IL-33 and TSLP release after allergen challenge [16].

Human observational data are inconsistent. Sdona et al. (2022), in the Swedish BAMSE cohort of 2285 children followed to age 24, reported a protective association between fibre intake at age eight and allergic rhinitis (OR 0.86 per 5 g/day; 95% CI 0.77–0.96) and IgE sensitization, but no significant association with asthma per se [42]. More decisively, in the first randomized trial of fibre supplementation in children with established asthma, Rank et al. (2026) administered 12 g/day of soluble corn fibre (≈50% of the recommended intake) for 4–6 weeks and found no significant differences in gut or nasal microbiome diversity, SCFA concentrations, or asthma symptom scores between intervention and placebo, though a trend toward increased *Bifidobacterium* was noted using compositionally appropriate differential abundance analysis (ANCOM-BC *p* = 0.0004, FDR q = 0.073) [27]. The authors concluded that higher doses, alternative fibre types, or selection of children with low baseline intake may be necessary, a finding that does not negate the mechanistic rationale but substantially raises the bar for trial design. Several factors may explain this null result; the 12 g/day dose may fall below the fermentative threshold needed to raise SCFA output meaningfully, the 4–6-week duration and probable limits on adherence may have been insufficient, and an unstratified population with established disease may dilute any benefit confined to specific endotypes or to earlier developmental windows. The trial is therefore best read as calibrating dose and design for future studies rather than as refuting the mechanistic rationale.

### 3.5. Dietary Timing: Does Protection Extend Beyond the Neonatal Window?

A recurring limitation of the literature is its focus on the first one to two years as the primary window of susceptibility, with insufficient attention to mid-childhood and adolescence. The critical-window hypothesis, while well supported for neonatal microbiome establishment, should not imply that dietary modification after age two carries no immunological consequence.

In the Avon Longitudinal Study of Parents and Children (ALSPAC), three dietary patterns identified at age seven, “processed”, “traditional”, and “health-conscious”, were followed to age 15.5 years with spirometry. A “health-conscious” diet in mid-childhood was independently associated with higher subsequent FEV^1^ and FVC, while a processed-food-rich diet was associated with lower lung function after adjustment for early-life exposures [43]. The mechanism may involve ongoing modulation of effector rather than regulatory lymphocyte populations; once Treg populations are established in infancy, circulating memory Th2 cell proportions may be more resistant to dietary modification than naive T cell polarization, which is uniquely sensitive to early-life SCFA gradients.

Prospective cohort data converge on the same gradient while exposing its limits. In the French PARIS cohort, high adherence to a Mediterranean diet at eight years was associated with reduced asthma and aeroallergen sensitization and with higher FEV1 and FVC at the same age, indicating that diet quality at school age tracks with concurrent respiratory health [44]. Whether such mid-childhood adherence alters the longer-term trajectory is less certain. In the Swedish BAMSE cohort, a Mediterranean-type diet at eight years showed no association with asthma incidence up to twenty-four years, although among children who already had asthma, higher adherence was related to modestly better lung-function indices [45]. The timing of exposure appears decisive rather than incidental. In the ALSPAC cohort, Bédard et al. (2020), analyzing maternal Mediterranean diet during pregnancy, found no protective association with asthma or atopy at seven to nine years, contrasting with the early-life and school-age signals and reinforcing the interpretation that the same dietary pattern exerts different effects depending on the developmental moment at which it is applied [46]. Taken together, these data support a model in which diet quality remains relevant across childhood but its capacity to reshape the underlying immune set-point is greatest early and attenuates thereafter.

Figure 3 depicts this developmental gradient. The largest and partly irreversible immunomodulation occurs during the first 1000 days, when naive CD4+ T-cell polarization is maximally plastic and most responsive to SCFA gradients; the modifiable fraction diminishes through early and mid-childhood as memory lymphocyte populations consolidate, with a possible second window of microbiome instability at puberty. The effect is best understood as diminishing but not absent, largest in the first 100 days, and progressively smaller though non-negligible thereafter. The negative fibre trial in children with established asthma (Rank et al. 2026) is consistent with this interpretation [27]. By school age, the fraction of the immune set-point amenable to dietary modification is smaller, although dietary quality remains worth pursuing for its broader, low-risk benefits.

## 4. Dysbiosis and Airway Inflammation: Cohort Evidence and Mechanisms

### 4.1. Prospective Cohort Studies and the Problem of Outcome Heterogeneity

Three large prospective birth cohorts have generated the most rigorous human data, but important differences in design, sequencing, and outcome definition limit the degree to which their results constitute independent replication (Table 1). CHILD followed 319 infants and phenotyped asthma using the Asthma Predictive Index and physician confirmation at age three. WHEALS defined its primary outcome as atopic wheeze at 12 months, according to parental report and skin-prick positivity, capturing predominantly early transient wheeze rather than confirmed asthma. PASTURE followed children to approximately six years using GINA-based physician diagnosis. These distinctions matter biologically; early transient wheeze, atopic wheeze, and school-age confirmed asthma are partially overlapping but distinct entities with different natural histories and potentially different microbiome-mediated pathways.

With these caveats, the cohorts converged on consistent signals while also revealing endotype-relevant heterogeneity. In CHILD, the FLVR-depleted high-risk infants who later developed asthma showed, via the corresponding murine model, a Th1/Th17-neutrophilic rather than Th2-eosinophilic inflammatory signature [4], a finding that anticipates the endotype distinction discussed below. WHEALS replicated the protective association of *Bifidobacterium* abundance with reduced atopic wheeze [47]. In the PASTURE study, Depner et al. (2020) demonstrated that microbiome maturation (modelled as microbiome age) at 12 months was inversely associated with school-age asthma (OR 0.72; 95% CI 0.56–0.93), and that fecal butyrate at 12 months independently predicted lower asthma risk (OR 0.28; 95% CI 0.09–0.91), directly linking microbial metabolic function to clinical outcome [48]. Convergent translational evidence comes from Ito et al. (2023), who showed in a murine model that neonatal intestinal propionate suppressed later airway inflammation via GPR41, and that fecal propionate measured at one month was reduced in infants who subsequently developed asthma [49].

### 4.2. Inflammatory Biomarkers and the Type-2/Non-Type-2 Distinction

The mechanisms described in Section 2, including SCFA-driven Treg induction suppressing Th2 responses, are most relevant to the type-2 (eosinophilic) asthma endotype, which predominates in atopic childhood asthma. FeNO reflects iNOS activity in the bronchial epithelium driven by IL-4 and IL-13 via JAK1/STAT6 and is the most accessible paediatric biomarker of type-2 inflammation [17]. Inverse associations between gut microbiome alpha diversity and FeNO have been reported cross-sectionally, though prospective same-cohort data measuring both variables remain scarce [17,50].

The non-type-2 (neutrophilic) endotype, by contrast, is driven by Th17 rather than Th2 responses and is comparatively corticosteroid-resistant. Here, the relevant microbial pathway is not principally SCFA–Treg–Th2 but the tryptophan–AhR–Th17 axis described in Section 2.3; microbial indole metabolites suppressing Th17 differentiation may modulate neutrophilic airway inflammation through mechanisms distinct from those governing eosinophilic disease. This framework gains support from a detail of the CHILD study itself; as noted in Section 2.4, FLVR supplementation in the murine model attenuated Th1/Th17 cytokines (IL-6, IL-17A, TNF) and neutrophilic infiltration rather than eosinophilia or IgE [4], suggesting that early microbial deficiency may predispose preferentially toward the non-type-2 axis. The endotype-specific mapping that includes type-2 disease responsive to the SCFA axis and non-type-2 disease potentially modifiable through AhR signalling has not been systematically tested but provides a coherent framework for interpreting otherwise divergent findings across asthma phenotypes; this is summarized in Figure 1. The inferential basis for this link is that AhR activation constrains the RORγt-driven transcriptional programme required for Th17 differentiation; a diet-driven reduction in indole ligands therefore removes a brake on Th17 responses, providing a plausible route by which low-fibre, dysbiotic states favour neutrophilic rather than eosinophilic inflammation. We note that this chain rests largely on experimental models and remains to be confirmed directly in paediatric airway disease.

Regarding IgE, Cahenzli et al. (2013) demonstrated in a murine model that diverse intestinal microbiota suppressed IgE by promoting IgA class-switching in germinal-centre B cells, a process abrogated in germ-free animals showing supranormal serum IgE [51]. Reduced Clostridiales diversity at 12 months predicted elevated total IgE and polysensitization at 36 months in CHILD [4]. Mahdavinia et al. (2023) identified reduced *Prevotella*, *Bifidobacterium breve*, and *Bifidobacterium catenulatum* in children with asthma versus controls, with these taxa accounting for measurably lower fecal SCFA concentrations [52].

### 4.3. Methodological Heterogeneity and the Limits of Causal Inference

The coherence of this evidence should not obscure its limitations, several of which are technical and consequential. Most cohort studies have employed 16S rRNA amplicon sequencing, which resolves taxonomy reliably only to genus level; species- and subspecies-level claims throughout the literature require metagenomic confirmation, and conflating the two overstates taxonomic certainty. Beyond the sequencing platform, multiple pre-analytic and analytic choices compromise cross-study comparability. The DNA extraction method introduces substantial bias, particularly against Gram-positive taxa such as *Faecalibacterium* with robust cell walls; the amplified variable region (V3–V4 versus V4) yields non-comparable taxonomic lists; and the reference database (SILVA, Greengenes, RDP) alters assignments. The field’s transition from operational taxonomic units (OTUs) to amplicon sequence variants (ASVs) has improved reproducibility but renders older and newer studies only partially comparable.

A further, under-appreciated issue is compositionality. Microbiome sequencing yields relative abundances constrained to a constant sum; so, conventional statistics (*t*-tests, Pearson correlations on relative abundances) generate spurious associations. Compositionally aware methods such as ANCOM-BC, ALDEx2, and centred log-ratio transformation, are now standard, and the recent trial by Rank et al. (2026) appropriately reports ANCOM-BC results [27]. Many earlier cohort analyses predate routine adoption of these methods, a consideration that should temper confidence in some historically reported taxon–asthma associations.

Reverse causation cannot be excluded. Asthma medications, inhaled and systemic corticosteroids, antibiotics, bronchodilators, independently alter gut microbiota. Corticosteroids impair mucosal secretory IgA, antibiotics acutely reduce diversity, and beta-agonists may shift fermentation by altering motility [53]. Confounding by socioeconomic status, pet ownership, and passive smoke exposure, all co-varying with both diet and microbiome, is inconsistently adjusted for across studies.

## 5. Specific Nutritional Factors: Evidence and Critical Appraisal

### 5.1. Omega-3 Long-Chain Polyunsaturated Fatty Acids

Omega-3 LC-PUFAs such as eicosapentaenoic acid (EPA) and docosahexaenoic acid (DHA) modulate gut microbiota while exerting direct anti-inflammatory effects on airway epithelium and immune cells. Supplementation increases the relative abundance of *Bifidobacterium* and *Lactobacillus* while reducing *Bacteroides fragilis* and *Ruminococcus gnavus*, a mucin-degrading species associated with barrier disruption and Th2 sensitization [54,55].

The pivotal clinical evidence is the COPSAC2010 trial. Bisgaard et al. (2016) randomized 736 pregnant women at 24 weeks of gestation to fish oil (2.4 g EPA + DHA/day) or olive oil placebo administered through the third trimester, following offspring to age five [56]. Persistent wheeze or asthma occurred in 16.9% of the fish oil group versus 23.7% of controls (adjusted HR 0.69; 95% CI 0.49–0.97; *p* = 0.035), a 30.7% relative reduction [56]. The effect was strongest in offspring of women in the lowest baseline EPA + DHA tertile (HR 0.46; 95% CI 0.25–0.83), suggesting biomarker-guided precision supplementation [56]. A meta-analysis of seven RCTs confirmed reduced childhood wheeze with prenatal omega-3 (pooled RR 0.80; 95% CI 0.64–0.99), though dose and timing heterogeneity limit generalizability [57].

### 5.2. Vitamin D: Barrier Function and Immune Modulation

Vitamin D regulates intestinal barrier integrity through induction of tight junction proteins and antimicrobial peptides (cathelicidin, β-defensin 2), and it modulates immune polarization via the nuclear vitamin D receptor (VDR) on T cells, dendritic cells, and the airway epithelium [58]. VDR signalling suppresses IL-4 and IL-13 and promotes regulatory dendritic cell IL-10 secretion. Vitamin D deficiency (serum 25(OH)D < 50 nmol/L) is associated with reduced gut microbiota diversity and lower *Bifidobacterium* abundance cross-sectionally, though directionality is confounded by outdoor activity co-determining both microbiome exposure and vitamin D synthesis [59].

Interventional data are less compelling than the mechanistic rationale predicts. A Cochrane meta-analysis by Martineau et al. (2016) found that vitamin D reduced the rate of asthma exacerbations requiring systemic corticosteroids (RR 0.63; 95% CI 0.45–0.88) but had no significant effect on lung function or FeNO [60]. Primary prevention has been tested more directly in two prenatal supplementation trials. The VDAART randomized trial (Litonjua et al. 2016) gave pregnant women at high familial risk 4400 IU/day versus 400 IU/day of vitamin D and reported a 6.1% lower incidence of asthma or recurrent wheeze at three years in the offspring of the higher-dose (4400 IU/day) group relative to the 400 IU/day group, a reduction that did not reach statistical significance in an arguably underpowered design [61]. A pre-specified combined analysis of VDAART and the COPSAC2010 vitamin D trial subsequently found a greater than 20% reduction in early-childhood asthma or recurrent wheeze, strongest among women with the lowest baseline 25(OH)D, mirroring the biomarker-dependent effect seen with omega-3 supplementation, as discussed in Section 5.1 [62]. As with the fibre and probiotic literature, however, the benefit attenuated with longer follow-up and was not framed around a microbiome endpoint. The microbiome-mediated component remains unmeasured in existing trials. The determinants of early-life vitamin D status are themselves nutritionally and developmentally complex; maternal supplementation during pregnancy shapes neonatal vitamin D stores, as shown in a Romanian prospective cohort of mother and preterm infant dyads [63], and the optimal timing and route of enteral supplementation in the most vulnerable infants remain unconfirmed [64]. These uncertainties compound the difficulty of isolating a microbiome-mediated effect, since vitamin D status, feeding modality, and microbial colonization co-vary from birth.

### 5.3. Human Milk Oligosaccharides and Prebiotic Supplementation

HMOs function as selective prebiotics rather than host nutrients. *B. longum* subsp. *infantis* possesses a comprehensive HMO-utilization gene cluster enabling dominance of the breastfed infant gut to a degree unachievable by formula-fed microbiomes receiving structurally distinct bovine oligosaccharides [29]. Downstream consequences include higher fecal IgA, reduced calprotectin, and, in translational murine studies, attenuated ovalbumin-induced airway inflammation.

Synthetic prebiotic mixtures (galacto-/fructo-oligosaccharides at 9:1, evaluated in the KOALA and GINI trials) are associated with modest reduction in early wheezing and atopic dermatitis in high-risk infants when given in the first six months, but effects on diagnosed asthma at school age are not sustained in most cohorts, possibly reflecting the transient nature of early microbiome effects once supplementation stops [65,66].

The clinical evidence for HMOs as formula ingredients, as distinct from the synthetic GOS/FOS prebiotics described above, has matured rapidly. In a foundational randomized multicentre trial, Puccio et al. (2017) fed healthy term infants a cow’s milk-based formula supplemented with 2′-fucosyllactose (2′-FL) and lacto-N-neotetraose (LNnT) and reported growth equivalent to a non-supplemented control, alongside fewer parent-reported episodes of bronchitis and lower respiratory tract infection and reduced antibiotic and antipyretic use through twelve months [67]. The microbial correlate was defined by Berger et al. (2020); infants receiving the two-HMO formula developed a faecal community type resembling that of breastfed infants, and carriage of this community type was associated with an approximately two-fold lower risk of requiring antibiotics in the first year [68]. These data position HMO-supplemented formula as the closest dietary approximation to the breastfeeding-driven ecology described in Section 3.2.

This rationale has since been extended to the extensively hydrolyzed formulas (eHF) used to manage cow’s milk protein allergy (CMPA); by definition, this population does not receive cow’s milk protein and carries an elevated risk of respiratory infection. The IVORY study (Nowak-Wegrzyn et al. 2019) first established that a whey-based eHF supplemented with 2′-FL and LNnT met American Academy of Pediatrics hypoallergenicity criteria, with 98.4% of children with CMPA tolerating the formula under double-blind, placebo-controlled food challenge [69]. The subsequent CINNAMON trial (Vandenplas et al. 2022) confirmed that this HMO-supplemented eHF, at reduced protein content, supported normal growth and controlled CMPA symptoms, while infants in the HMO arm had significantly fewer upper respiratory tract infections and a lower risk of otitis media [70]. Boulangé et al. (2023), reporting for the CINNAMON investigators, found that adding the two HMOs to the eHF enriched faecal bifidobacteria and mechanistically delayed the shift of the microbiome toward an adult-like configuration, with concordant changes in amino acid degradation and bile acid conjugation that were most pronounced when supplementation began before three months of age [71]. The incorporation of HMOs into hypoallergenic hydrolysates thus represents a translational endpoint of the gut–lung axis logic of this review; even in infants who cannot be breastfed and require an extensively hydrolyzed diet, restoring a single class of milk oligosaccharide measurably moves the microbiome toward the configuration associated with respiratory protection. Whether this extends to reduced asthma incidence, as opposed to reduced early infection, has not yet been tested in an adequately powered trial.

### 5.4. Probiotics: Strain Specificity and the Evidence Ceiling

Probiotic supplementation with *Lactobacillus rhamnosus* GG, *L. reuteri* DSM 17938, and *Bifidobacterium lactis* Bb12, has been evaluated in multiple paediatric trials for asthma and wheeze prevention. The evidence base does not currently support clinical recommendations for asthma prevention. Pelucchi et al. (2012) found no significant effect of prenatal or postnatal probiotics on asthma or wheeze at school age [72], and a Cochrane review confirmed absent consistent benefit for respiratory outcomes while acknowledging reduced atopic dermatitis with specific strains [73].

The World Allergy Organization position paper on Clinical Use of Probiotics in Pediatric Allergy (CUPPA), authored by Fiocchi et al. (2016), reached a concordant conclusion for asthma, while noting a conditional signal for eczema prevention in high-risk infants, and it stressed that evidence quality across indications was low to very low by GRADE criteria [74]. This institutional consensus should anchor any future probiotic trial design for respiratory outcomes. The fundamental problem is strain specificity; different *Lactobacillus* strains produce distinct immunological effects, some Th1-promoting, others Treg-expanding, and the categorical label “probiotics” obscures these distinctions. Future trials require strain-specific protocols, post-supplementation engraftment confirmation by sequencing, and mechanistic substudies measuring SCFA production and Treg induction. Critically, probiotics should not be regarded as a homogeneous intervention. Pooling heterogeneous strains in meta-analyses obscures effects that may be strain-specific, and future clinical studies should evaluate defined single strains against defined outcomes rather than aggregate formulations.

Attention to strain identity extends beyond bacteria to probiotic yeasts, which the preceding discussion does not address. Saccharomyces boulardii, a non-colonizing yeast mechanistically distinct from bacterial probiotics, is intrinsically resistant to gastric acid and to antibacterial antibiotics, transits the gut without permanent engraftment, and exerts its effects through secreted factors rather than through lasting shifts in community composition. Preclinical work indicates that it can modulate the gut microbial ecosystem, reinforce epithelial tight-junction integrity, and attenuate systemic and mucosal inflammatory signalling, and clinical evidence supports its use in paediatric gastrointestinal conditions such as antibiotic-associated and infectious diarrhoea. Its relevance to allergic airway disease, however, remains hypothesis-generating. Direct paediatric asthma data are lacking, and its position with respect to the gut–lung axis is mechanistically plausible but not yet demonstrated in respiratory outcomes. We include it here to signal that the evidence gap relating to probiotics encompasses fungal as well as bacterial agents, and that trials of defined yeast strains represent an under-explored avenue.

### 5.5. Polyphenols and Antioxidants

Dietary polyphenols such as quercetin, resveratrol, luteolin, and anthocyanins undergo limited small-intestinal absorption but are extensively metabolized by colonic bacteria, exerting bidirectional effects on composition [75]. *Akkermansia muciniphila* and *Faecalibacterium prausnitzii* are selectively promoted by polyphenol-rich substrates in ex vivo fermentation models. In paediatric asthma epidemiology, antioxidant intake shows inconsistent associations when analyzed independently of dietary pattern, because polyphenol-rich diets co-deliver more fibre and omega-3 fatty acids, making independent attribution intractable [76]. Controlled paediatric data with microbiome and FeNO endpoints are insufficient for recommendations.

## 6. The Obesity-Related Asthma Phenotype

Any account of diet, microbiota, and paediatric asthma that omits the obesity-related phenotype is incomplete, because this phenotype sits precisely at the intersection of the three. Obesity-related asthma is clinically and immunologically distinct from classical atopic asthma. It is associated with more severe manifestations, poorer disease control, reduced corticosteroid responsiveness, and, mechanistically, a shift away from the Th2/eosinophilic profile toward Th1- and Th17-polarized, non-type-2 inflammation [77]. Hu et al. (2023) reviewed the mediating role of gut microbiota in this phenotype, arguing that microbial dysbiosis links the two conditions by influencing host lipid metabolism, triggering chronic low-grade systemic inflammation, and modulating immune responses [77]. This non-type-2 signature connects directly to the mechanistic framework discussed in Section 2.1; the same Th17-polarised, corticosteroid-resistant profile produced experimentally when FLVR taxa were transplanted into germ-free mice (Arrieta et al.) is recapitulated in obesity-associated dysbiosis, and the tryptophan–AhR–Th17 axis provides the shared molecular link. A Western dietary pattern, high in saturated fat and low in fermentable fibre, is the proximate driver. It depletes SCFA-producing and indole-generating commensals, lowers butyrate and AhR-ligand availability, and thereby releases the Th17 programme from microbial restraint, producing the neutrophilic, non-atopic inflammation that characterizes this phenotype. Obesity-related asthma is thus not a separate track but the clinical expression of the non-type-2 route discussed earlier in this review.

The microbial signature of obesity in children, an increased Firmicutes-to-Bacteroidetes ratio and reduced overall diversity, overlaps with the dysbiotic pattern associated with asthma risk, suggesting shared ecological determinants. Critically for a nutritional review, this has direct implications for dietary intervention. A child with obesity-related asthma carries a different baseline microbiome, a different inflammatory endotype, and potentially a different response to fibre or omega-3 supplementation than a norm-weight atopic child. The dose-finding negative result of Rank et al. (2026) takes on additional meaning in this light, as intervention effects may be modified by adiposity-associated baseline dysbiosis, an interaction that no paediatric fibre trial has yet been powered to detect [27]. Paediatric obesity has risen across most world regions over recent decades [77], and early feeding practices, maternal weight, and the protective role of breastfeeding are recognized modifiable determinants of later adiposity, including in Romanian cohorts [78]; this phenotype therefore warrants explicit consideration in any nutritional prevention strategy.

## 7. Clinical Implications and Research Priorities

### 7.1. Translating Evidence into Paediatric Practice

The evidence supports a model in which early-life dietary quality is a modifiable determinant of gut microbiota composition, which shapes the immunological risk architecture for paediatric asthma. Nutritional counselling during pregnancy and early life, currently focused on macronutrient adequacy and micronutrient supplementation, should be extended to encompass dietary diversity, fermentable fibre intake, omega-3 PUFA consumption, breastfeeding support, and counselling on the prevention and management of childhood overweight. The strength of evidence, however, differs substantially across these interventions and should be communicated accordingly (Table 2).

Breastfeeding support rests on the strongest prospective and mechanistic evidence; prenatal omega-3 supplementation has one large RCT (COPSAC2010) and a supporting meta-analysis, making it the most actionable single nutritional intervention; dietary fibre and Mediterranean-pattern counselling are supported by consistent observational associations but lack interventional proof in children, and the first fibre RCT was negative at the dose tested. This hierarchy should guide clinical communication: breastfeeding and prenatal omega-3 can be recommended with reasonable confidence; fibre and dietary diversity are appropriately framed as beneficial according to existing evidence, without known risk; specific probiotic strains for asthma prevention are not justified by current evidence.

In paediatric allergology practice, a nutritional history covering breastfeeding duration, complementary food introduction, current fibre intake, ultra-processed food consumption, and, critically, adiposity status provides mechanistic context for the observed inflammatory phenotype and identifies families amenable to low-risk modification. This is particularly salient in Central and Eastern European populations, where the documented east–west gradient in both dietary patterns and asthma prevalence, combined with rising paediatric obesity, defines a distinct clinical context that the predominantly Western European and North American evidence base does not fully capture. Brief structured dietary screening integrated into routine atopic child follow-up would allow systematic identification of high-risk profiles, analogous to FeNO measurement as a standard biomarker of type-2 inflammation [79].

### 7.2. Gaps Limiting Causal Inference

Several gaps prevent translation into definitive guidelines. In the first adequately powered fibre RCT in children with asthma, Rank et al. (2026) reported negative results at the dose tested, indicating the hypothesis is not falsified but the parameters of effective intervention remain undefined [27]. Higher doses, longer supplementation, fibre types with greater fermentation specificity, and selection of children with demonstrably low baseline diversity all merit systematic evaluation. Dietary assessment carries substantial measurement error, particularly for ultra-processed foods, increasingly implicated in microbiota disruption through emulsifiers (carboxymethylcellulose, polysorbate 80) and artificial sweeteners (sucralose).

The gut microbiome’s profound inter-individual variability limits cross-population generalization. Taxa associated with protection in Canadian and European cohorts may not occupy equivalent functional niches in East Asian, African, or South American children, where baseline composition, traditional diets, and asthma phenotypes differ [80]. Residual confounding from socioeconomic status, pet ownership, and passive smoke exposure remains inadequately addressed in most observational work, and the obesity–microbiome–asthma interaction (Section 6) compounds this by introducing adiposity as both confounder and effect modifier.

### 7.3. Research Priorities and the Adolescent Gap

Three directions appear productive. The first of these points towards RCTs of prebiotic fibre, defined substrates (arabinoxylan, long-chain inulin) at doses calibrated above the threshold established by Rank et al. (2026), with gut microbiota composition, fecal SCFA, Treg frequency, and FeNO as pre-specified endpoints, stratified by baseline microbiome, fibre intake, adiposity, and inflammatory endotype (type-2 eosinophilic versus non-type-2) [27]. Second, prospective multi-omic cohort studies should integrate longitudinal diet with shotgun metagenomics, metabolomics (SCFA, bile acid, tryptophan-metabolite profiling), and immunophenotyping to reconstruct the causal pathway in individual children. Third, research would benefit from trials of Mediterranean-pattern dietary counselling in pregnant atopic women, with offspring gut microbiota at three months as a primary outcome and asthma at five years as secondary [28].

A conspicuous gap is present with regard to adolescence. The gut microbiome undergoes a second period of instability associated with puberty-related hormonal shifts, changing diet, antibiotic exposure, and lifestyle changes affecting microbial structure [81]. Whether the microbiome contributes to the female-predominant post-pubertal shift in asthma prevalence is an intriguing, insufficiently investigated hypothesis [82]. Extending microbiome research and dietary trials into adolescence is essential if the gut–lung axis is to be understood as a dynamic, lifelong mechanism rather than a neonatal phenomenon.

## 8. Conclusions

The relationship between diet, gut microbiota, and paediatric asthma is mechanistically coherent, epidemiologically consistent across multiple large prospective cohorts, and biologically plausible. A chain of evidence, dietary substrate → microbial fermentation → SCFA production → Treg induction → attenuated Th2 airway inflammation, is supported by preclinical data of considerable rigor, prospective cohort associations of meaningful effect size, and early interventional signals consistent with the proposed mechanism, while remaining endotype-specific. The evidence is most applicable to type-2 eosinophilic disease, with the tryptophan–AhR–Th17 axis offering a parallel framework for non-type-2 and obesity-related phenotypes.

The first human dietary fibre RCT in children with asthma (Rank et al. 2026) produced negative results at the dose tested, underscoring that mechanistic plausibility does not automatically translate into clinical efficacy at conventional doses [27]. The next decade should prioritize dose-escalation and fibre-type trials with microbiome engraftment as a primary endpoint, embed compositionally appropriate multi-omic measurement in prospective cohorts, account for adiposity as effect modifier, ensure cross-cultural replication, and extend investigation from the neonatal period through adolescence.

Until that evidence base is consolidated, promoting dietary diversity, adequate fermentable fibre intake, breastfeeding support, counselling on the prevention and management of childhood overweight, and Mediterranean-style dietary patterns in paediatric and obstetric practice remains justified by the existing evidence, carries no risk of harm, and aligns with general nutritional guidance for children.

## Figures and Tables

**Table 1:** Comparison of the three principal prospective birth cohorts informing the gut microbiota–asthma association. Differences in sequencing approach and outcome definition limit direct cross-cohort comparison. API, Asthma Predictive Index; CI, confidence interval; FLVR, *Faecalibacterium*–*Lachnospira*–*Veillonella*–*Rothia*; GINA, Global Initiative for Asthma; SPT, skin-prick test.

Cohort	Country/N	Sequencing	Asthma Outcome	Key Microbiome Finding
CHILD	Canada; 319 infants	16S rRNA (V4)	API + physician diagnosis at 3 y	FLVR depletion in first 100 d; high-risk (wheeze + atopy) OR 21.5 for asthma at 3 y. Murine model: Th1/Th17-neutrophilic, not Th2
WHEALS	USA; ~1258 children	16S rRNA	Atopic wheeze at 12 mo (parental report + SPT)	Reduced *Bifidobacterium* at 1 mo; effect attributed to *B. longum* subsp. *infantis*
PASTURE	Europe; 618–1133 infants	16S rRNA	GINA-based physician diagnosis at ~6 y	Microbiome maturation inversely linked to asthma (OR 0.72); fecal butyrate protective (OR 0.28, 95% CI 0.09–0.91)

**Table 2:** Nutritional interventions for paediatric asthma prevention, graded by strength of current evidence. The gradient from breastfeeding (strongest) to probiotics and polyphenols (insufficient) should guide clinical communication. The downward arrow (↓) denotes a reduction in the stated outcome. EPA, eicosapentaenoic acid; DHA, docosahexaenoic acid; HMO, human milk oligosaccharide; HR, hazard ratio; IRR, incidence rate ratio; OR, odds ratio; RCT, randomized controlled trial; WAO, World Allergy Organization.

Intervention	Strength of Evidence	Observed Effect	Key Reference(s)
Breastfeeding	High (prospective cohorts + mechanistic)	↓ early wheezing; HMO-driven *Bifidobacterium* dominance	Bode 2012 [29]; Roduit 2014 [30]
Prenatal omega-3 (EPA + DHA)	Moderate–high (1 large RCT + meta-analysis)	30.7% ↓ persistent wheeze/asthma (HR 0.69)	Bisgaard 2016 [56]; Zhang 2021 [57]
Mediterranean dietary pattern	Moderate (consistent observational; high I^2^)	↓ current & severe wheeze (OR 0.85/0.66)	Garcia-Marcos 2013 [23]; Kouvari 2022 [24]
Dietary fibre/prebiotics	Low–moderate (1 RCT negative at tested dose)	Mechanistic support; no clinical effect at 12 g/day	Trompette 2014 [7]; Rank 2026 [27]
Vitamin D	Moderate (for exacerbations only)	↓ exacerbations needing steroids (IRR 0.74); no FeNO effect	Martineau 2016 [60]
Probiotics	Low/insufficient (WAO: low-very low GRADE)	No consistent asthma benefit; eczema signal only	Pelucchi 2012 [72]; Fiocchi 2016 [74]
Polyphenols/antioxidants	Insufficient in children	Promotes *Akkermansia*, *F. prausnitzii* ex vivo; confounded epidemiology	Duda-Chodak 2012 [75]; Hosseini 2017 [76]

## Data Availability

No new data were created or analyzed in this study. Data sharing is not applicable to this article.

## Abbreviations

The following abbreviations are used in this manuscript:

AhR	aryl hydrocarbon receptor
ALDEx2	ANOVA-like differential expression analysis
ALSPAC	Avon Longitudinal Study of Parents and Children
ANCOM-BC	analysis of composition of microbiomes with bias correction
API	Asthma Predictive Index
ASV	amplicon sequence variant
BAMSE	Swedish birth cohort (Barn/Children, Allergy, Milieu, Stockholm, Epidemiology)
BCL6	B-cell lymphoma 6
CD4	cluster of differentiation 4
CHILD	Canadian Healthy Infant Longitudinal Development study
CI	confidence interval
CLR	centred log-ratio
CNS1	conserved non-coding sequence 1
CUPPA	Clinical Use of Probiotics in Pediatric Allergy
DC	dendritic cell
DHA	docosahexaenoic acid
DNA	deoxyribonucleic acid
EFRAIM	Mechanisms of Early Protective Exposures on Allergy Development study
EFSA	European Food Safety Authority
EPA	eicosapentaenoic acid
FDR	false discovery rate
FEV1	forced expiratory volume in one second
FeNO	fractional exhaled nitric oxide
FFAR2/FFAR3	free fatty acid receptor 2/3 (GPR43/GPR41)
FLVR	*Faecalibacterium*–*Lachnospira*–*Veillonella*–*Rothia*
FOXP3	forkhead box P3
FVC	forced vital capacity
GA2LEN	Global Allergy and Asthma European Network
GAN	Global Asthma Network
GATA3	GATA-binding protein 3
GINA	Global Initiative for Asthma
GINI	German Infant Nutritional Intervention study
GPR41/GPR43/GPR109a	G-protein-coupled receptor 41/43/109a
GRADE	Grading of Recommendations Assessment, Development and Evaluation
HCAR2	hydroxycarboxylic acid receptor 2 (GPR109a)
HDAC	histone deacetylase
HIF	hypoxia-inducible factor
HMO	human milk oligosaccharide
HR	hazard ratio
IgA	immunoglobulin A
IgE	immunoglobulin E
IL	interleukin (IL-4, IL-5, IL-6, IL-8, IL-10, IL-13, IL-17A, IL-22, IL-33)
iNOS	inducible nitric oxide synthase
IRR	incidence rate ratio
ISAAC	International Study of Asthma and Allergies in Childhood
JAK1	Janus kinase 1
KOALA	Dutch birth cohort (Child, Parent and Health: Lifestyle and Genetic Constitution)
LC-PUFA	long-chain polyunsaturated fatty acid
LPS	lipopolysaccharide
MHC-II	major histocompatibility complex class II
NOD2	nucleotide-binding oligomerization domain-containing protein 2
OR	odds ratio
OTU	operational taxonomic unit
PASTURE	Protection Against Allergy: Study in Rural Environments
RCT	randomized controlled trial
RDP	Ribosomal Database Project
SCFA	short-chain fatty acid
SILVA	ribosomal RNA database (not an acronym)
SPT	skin-prick test
STAT6	signal transducer and activator of transcription 6
TGF-β	transforming growth factor beta
TGR5	Takeda G-protein receptor 5
Tfh	T follicular helper (cell)
Tfh13	T follicular helper cell subset 13
Th1/Th2/Th17	T helper cell type 1/2/17
TLR	Toll-like receptor (TLR2, TLR4)
TNF	tumour necrosis factor
Treg	regulatory T cell
TSLP	thymic stromal lymphopoietin
VDR	vitamin D receptor
WHEALS	Wayne County Health, Environment, Allergy and Asthma Longitudinal Study
WAO	World Allergy Organization
ZO-1	zonula occludens-1
